# On the Adhesive Interaction Between Metals in Atomistic Simulations of Friction and Wear

**DOI:** 10.1007/s11249-024-01865-1

**Published:** 2024-06-24

**Authors:** Mohammad Aramfard, Luca Avanzi, Lucia Nicola

**Affiliations:** Department of Industrial Engineering, University of Padova, 35131 Padua, Italy

**Keywords:** Adhesive wear, Atomistic simulation, Interatomic potentials, Contact

## Abstract

Atomistic simulations are performed to assess how the main characteristics of a pairwise interatomic potential function can affect the occurrence of wear. A Morse-like potential is tailored in its attractive part such as to vary independently the cut-off radius and the maximum value of the attractive (adhesive) force. An ideal numerical experiment is then performed where the interaction between a metal crystal and a probe changes, while their material properties are not affected, to isolate the behavior of the interface. Force functions with larger adhesive force can loosely be interpreted as describing dry contacts while those with smaller adhesive force can be interpreted as describing lubricated contacts. Results demonstrate that the occurrence of wear is strongly dependent on the shape of the interatomic force field, and more specifically on the combination of maximum adhesive force and effective length of the interatomic attraction. Wear can initiate also at small adhesive energy, provided that the maximum adhesive force between atoms is large. When the surface of the crystal is taken to be rough instead of flat, the effect of the interatomic potential function on friction and wear becomes smaller, as the atoms belonging to the roughness are weakly bound to the rest of the crystal and are easily dislodged with any of the force functions we used.

## Introduction

In daily life, hard solids pressed into contact do not stick to each other. This is different at the atomic scale, where adhesive forces are significant and can affect the frictional and wear behavior of metal components in relative motion. Two main adhesive wear mechanisms have been proposed in the literature: fracture-induced wear, by Archard in 1953 [1], and progressive smoothing of surface asperities, by Holms in 1976 [2]. Much later, both mechanisms have been observed experimentally by means of an atomic force microscope [3–5] or a transmission electron microscope [6, 7]. Also atomistic simulations have shown both behaviors, the smoothing of asperities by means of atomic wear [8–11], and fracture with the formation of debris [12].

Rabinowicz [13] was the first to bring forward the idea that the occurrence of fracture in adhesive contacts would depend on the size of the contacting asperities. This has been confirmed recently by Aghababaei et al. [12] by means of atomistic simulations. Using dedicated interatomic potential functions, they demonstrated that for a given material, below a critical asperity size, wear occurs through smoothening of the asperity, above that size, through fracture of the asperity, and formation of debris. In the latter case, after formation of the third body, a steady state would establish, in line with experimental observations. It appears that the first molecular dynamics simulations of contact could not capture fracture, just because they were performed on a too small scale.

In the study by Aghababaei et al. [12], a modified pairwise interatomic Morse potential [14] was used to describe the interatomic interaction inside the bodies in contact as well as their adhesive properties. The compressive behavior of the solids was kept unchanged, as only the tail of the potential was modified while the depth of the potential well and the repulsive interaction were kept unaltered. Any change in the tail of the potential affects concurrently the range of interaction between the solids, and the response of the atoms under tension. A change in the potential reflects, thus, not only on the attraction between bodies, but also on a change in the properties of the material, as for instance its unstable stacking fault energy, by that affecting the onset of plasticity in the crystal and its propensity to fail in a brittle or ductile manner. Following an approach similar to Aghababaei et al. [12], Alhafez and Urbassek [15] used a modified Lennard–Jones potential to show that increasing the strength of adhesion increases the pile-up in front of a rigid probe scratching a Fe block. Also in this work, the response on compression of Fe and its plastic behavior are affected by the change in adhesive strength, so it is difficult to isolate the cause for the increase in the pile-up size.

Here, we intend to study how adhesion influences atomic scale wear in small contact asperities, those which do not behave brittle, by isolating the effect of adhesion from that of material properties. By only considering interfacial adhesion and varying the shape of the potential function, we can investigate the relative relevance of the maximum attractive force and the range of attraction (the length above which the attractive forces vanish) on atomistic wear. To this end, we model adhesion between a rigid probe and a metal crystal by means of a modified Morse potential, while we model the metal crystal by a standard EAM potential for copper. This model intends to mimic the adhesive properties of a counterbody, the probe, without explicitly modeling a real lubricant, adhesive layer, or tribolayer between the contacting bodies.

Instead of performing classical atomistic simulations, we carry out simulations using a dual-scale atomistic-dislocation dynamics framework [16] which agrees with atomistic simulations, and can tackle larger domains at the same computational cost. In this way, we avoid the approximations related to coarse-graining, as well as the excessive constraint that is typical of atomistic simulations that intend to mimic only a slice of a larger metal body. Specially in contact problems, when dislocations nucleate under the contact and glide towards the bottom of the domain they experience a constraint from the atoms at the bottom of the crystal which are fixed and, thus, repel the dislocations that would, in a larger body, glide freely.

## Problem Definition

**Fig. 1:**
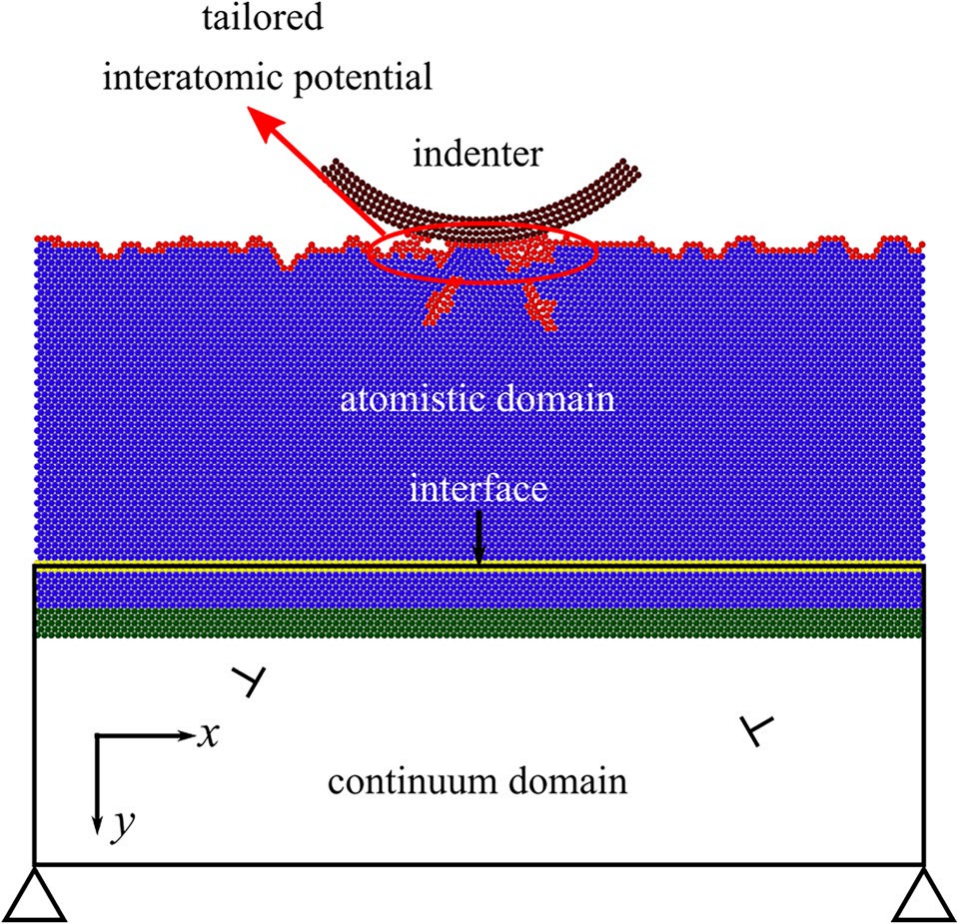
The section of a rigid cylindrical indenter enters into contact with a single crystal. The indenter and the top of the crystal are described atomistically, while the bottom of the crystal is described through a continuum, where dislocations can glide according to dislocation dynamics

A cylindrical probe first indents the crystal to the depth corresponding to a prescribed load and subsequently displaces horizontally with a tangential displacement up to 0.5Å. The problem, schematically represented in Fig. 1, is studied in two dimensions, considering plane strain conditions in the third dimension. The indenter is modeled as the section of a rigid hollow cylinder with outer radius $R={8}{\hbox {nm}}$. It has the same FCC crystalline structure as the substrate, but the atoms are very slightly displaced such as to obtain a cylindrical profile with smooth surface, i.e., without atomic steps. The spacing between the atoms in the probe is, thus, very similar to that of the atoms in the substrate.

The simulations are performed at 0K, to tackle only mechanical wear and exclude contributions from heat.

To study the effect of adhesive interaction between the indenter and the crystal, a new potential function based on the Morse potential [14] is derived which enables one to tailor the adhesive part of the potential as explained in Sect. 3. Following Aramfard et al. [16], the copper crystal is described using two domains modeled at different scales: while the upper part of the crystal is modeled atomistically, the bottom part is modeled as a linear elastic continuum domain, where dislocations that are nucleated in the atomistic domain can continue to glide according to constitutive rules, fitted to the atomistics. In these simulation, however, the loads applied are too small to drive dislocations to the continuum domain, and they can only be found in the atomistic part. The Embedded Atom Method (EAM) [17] many-body interatomic potential parametrized by Mishin et al. [18] for Cu is used for the interactions between atoms in the atomistic crystal. In lieu with previous studies [16, 19–21], and the Cu crystal is taken to have lattice parameter $a_0 = {2.46}$Å and to be oriented with its (111) plane in the *x,y* plane of analysis. The *x*-direction, where periodic boundary conditions are imposed, corresponds to the crystallographic $[\bar{1} 1 0]$ direction and the *y*-direction corresponds to the $[\bar{1} \bar{1} 2]$ direction. The top surface of the crystal is taken to be either flat or rough. At each incremental displacement of the indenter, static equilibrium is achieved by means of an iterative procedure: first the atomistic crystal is loaded with its bottom fixed at a tentative equilibrium position, and subsequently the continuum is deformed through the displacement computed near to the bottom of the atomistic domain. The new iterative step employs the displacements of the continuum domain to load the atomistic domain at its bottom, while the indenter kept fixed. The iterations continue until the difference in the potential energy of the atomistic domain, is smaller than a threshold for two subsequent iterations. The energy is minimized using the conjugate gradient method at ${0}{\hbox {K}}$ by means of the Large-scale Atomistic/Molecular Massively Parallel Simulator (LAMMPS). Details of the procedure and the validation against full atomistic simulations can be found in Aramfard et al. [16]. In the atomistic domain, the atomic stress is computed using the virial formulation [22], which provides the correct stress state in the solid, provided that one computes the volume of the two-dimensional solid taking into account that it is only one atom thick.

## The Form of the Interatomic Potential

**Fig. 2:**
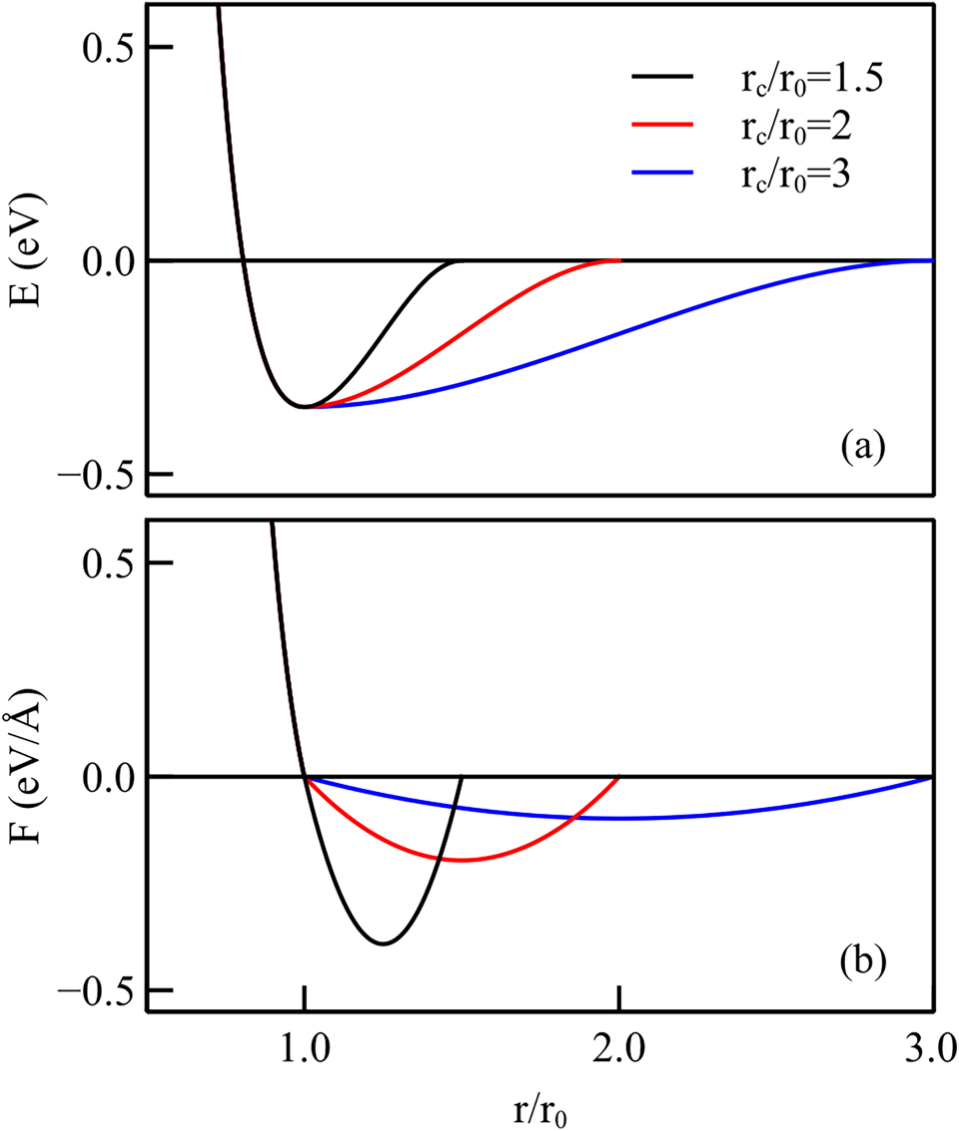
**a** Interatomic potential energy and **b** the corresponding interatomic force for the modified Morse potential with various values of the cut-off distance, according to the formulation proposed by Aghababaei et al. [12]

The pairwise Morse interatomic potential [14] is selected to describe the interaction between indenter and crystal because of its simple form, which permits one to correlate the shape of the potential to its effect on the simulations. In this work, we propose a new form of the interatomic Morse potential to tailor the adhesive part of the interaction. The work is inspired by that of Aghababaei et al. [12] who proposed to replace the adhesive part of the interaction in the Morse potential by a polynomial function, in order to vary the long-range interaction by keeping the short range interaction unchanged. In their work, the long-range interaction was modified by changing the cut-off distance, as shown in Fig. 2a. Evidently, such a change also affects the adhesive interaction between the bodies and consequently the stress distribution in the region just below the contact. Indeed, a different cut-off implies a rather significant change in the forces exchanged by the atoms in adhesive contact, as illustrated in Fig. 2b, where three Morse potential functions with exponential decay are cut-off at a distance of 1.5, 2, and 3 $r_\textrm{0}$. It is evident that increasing the range of adhesive interaction occurs at the cost of a weaker interaction. Note that the maximum of the adhesive force as well as the interatomic distance at which it occurs change. So there are two characteristic lengths that change in the problem: the length at which the attraction is negligible that we will call in the following the *range of attraction*, and the length at which the attraction is maximum. In addition, the curve representing the force has a non-smooth transition at 1.1 $r_0$ (where the deviation from the Morse potential is in place), as the derivatives are discontinuous there. This entails that if the interaction inside a material is described through such a potential, there is a sudden change of properties that are based on the second derivative of the potential energy, such as the elastic modulus, when the material is stretched above the corresponding strain.

To improve on the smoothness of the force function and on the flexibility of the potential function, we propose a new form of the interatomic Morse potential, which permits us to independently change: (1) the cut-off distance of the potential, (2) the maximum value of the adhesive force, $F_\textrm{max}$, and (3) the interatomic distance at which it occurs $r_\mathrm{F_\textrm{max}}$. The derivatives of the force function are enforced to be continuous to avoid sudden changes of interaction. In this way, we can assess what is the impact of each parameter on the atomic wear behavior of the surface.

The first part of the potential function up to the potential well at $r_0$ is kept identical to the original Morse potential and for $r > r_0$, an exponential form is proposed:

1 $$E(r) = \left\{ {\begin{array}{*{20}{lr}} {D_{0} [e^{{ - 2\alpha _{0} (r - r_{0} )}} - 2e^{{ - \alpha _{0} (r - r_{0} )}} ],} \hfill & {r < r_{0} } \hfill \\ {\sum\limits_{{i = 1}}^{5} {c_{i} } e^{{ - \alpha _{i} (r - r_{0} )}} }, \hfill & {r_{0} \le r < r_{c} } \hfill \\ \end{array} } \right.$$

where $D_0$, $\alpha _0,$ and $r_0$ are the usual Morse potential parameters to which we assign the values corresponding to Cu–Cu interaction [23]: $r_0 = {2.6260}$Å^1^, $D_0 = {0.3429}{\hbox {eV}},$ and $\alpha = 1.3588$Å. The cut-off distance is $r_c$. The $\alpha _i$ are the additional parameters introduced to modify the shape of the adhesive part of the potential and are defined as follows:

2 $$\left\{ {\begin{array}{*{20}l} {\alpha _{i} = \frac{\beta }{{d^{{i - 1}} }}} \hfill \\ {\beta = \frac{{\alpha _{0} }}{f}} \hfill \\ \end{array} } \right.$$

Here, *f* and *d* are constants introduced to control the minimum value of the force, i.e., the maximum adhesive force, $F_\textrm{max}$, and the interatomic distance at which the force is maximum, $r_\mathrm{F_\textrm{max}}$. Throughout this article, $F_\textrm{max}$ is reported in absolute value.

**Fig. 3:**
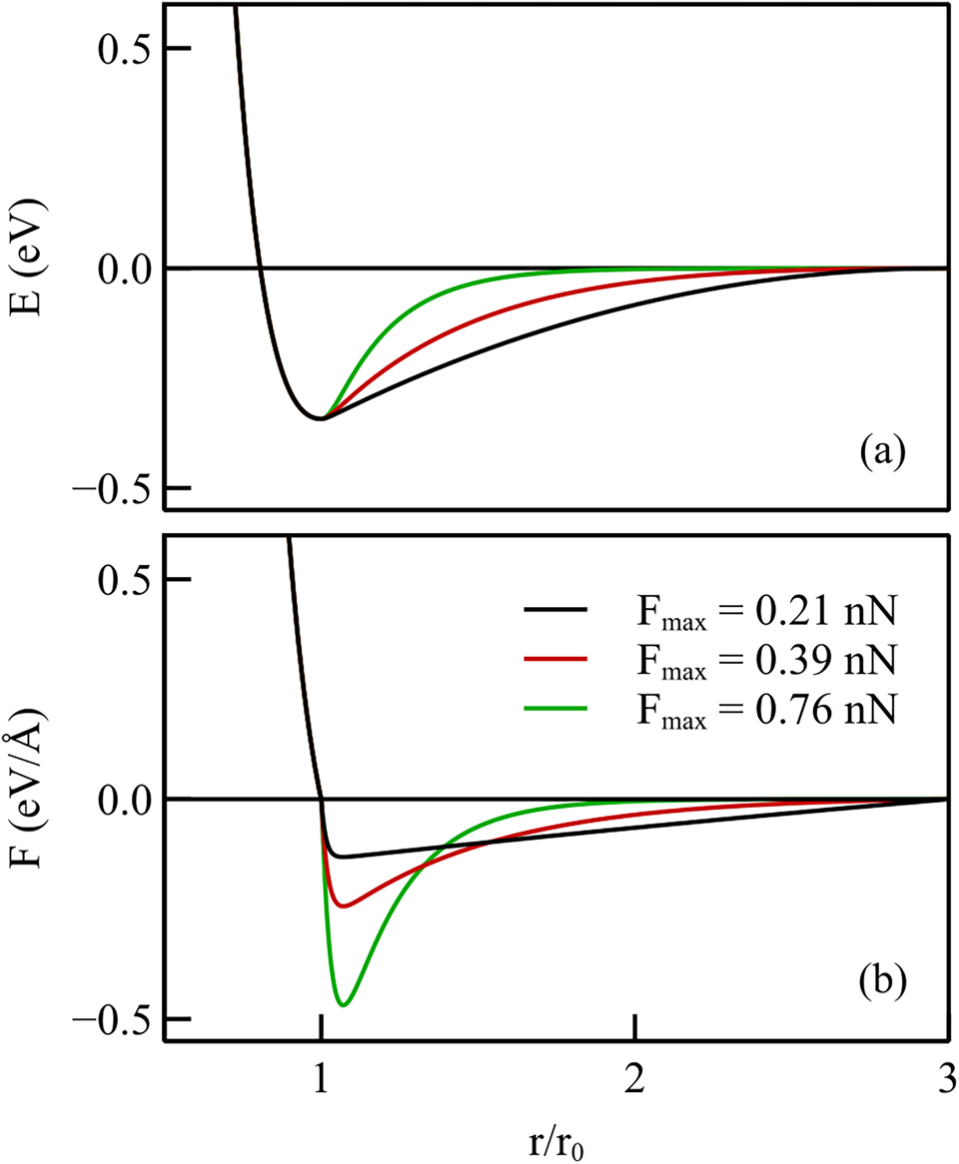
**a** Interatomic potential energy with the newly proposed potential, representing copper up to the minimum of the potential well, and having various shapes for the attractive part of the potential but the same cut-off radius; **b** the corresponding force distributions with different $F_\textrm{max}$

The coefficients $c_i$ in Eq. 1 are determined by means of five boundary conditions on the energy: *E*(*r*) is enforced to be continuous up to its second derivative at $r=r_0$ and *E*(*r*) and its first derivative are enforced to be zero at $r=r_c$. In addition to the continuity of the energy at $r=r_0$, the continuity of the first derivative ensures continuity of the force, defined by $F(r)=-E'(r)$ and the continuity of the second derivative ensures that the slope of the force is the same on the left and right sides of $r=r_0$. However, it is noted that due to numerical limitations, continuity is not strongly enforced when $r_\mathrm{F_\textrm{max}}$ is close to $r_0$ or $F_\textrm{max}$ is very large. This is not expected to have any consequences on the results considering that the potential here only describes adhesion, and has no influence on material properties. Figure 3a shows three different potential functions having the same cut-off radius, but slightly different shape, as they decay with different slope from the minimum, and Fig. 3b the corresponding force curves. Note that the different potentials, albeit having the same cut-off, correspond to force–distance curves with significantly different shape, including different values for the maximum of the attractive force. In the following, we will show what is the effect of the cut-off radius and of the maximum adhesion force on the response of the metal crystal to the sliding probe. In Section 4, we first investigate how friction and wear are affected by a simultaneous change of $r_\textrm{c}$ and $F_\textrm{max}$, and then we consider the independent change of the two parameters.

## Interatomic Potential Functions with Various Cut-off Radii

**Fig. 4:**
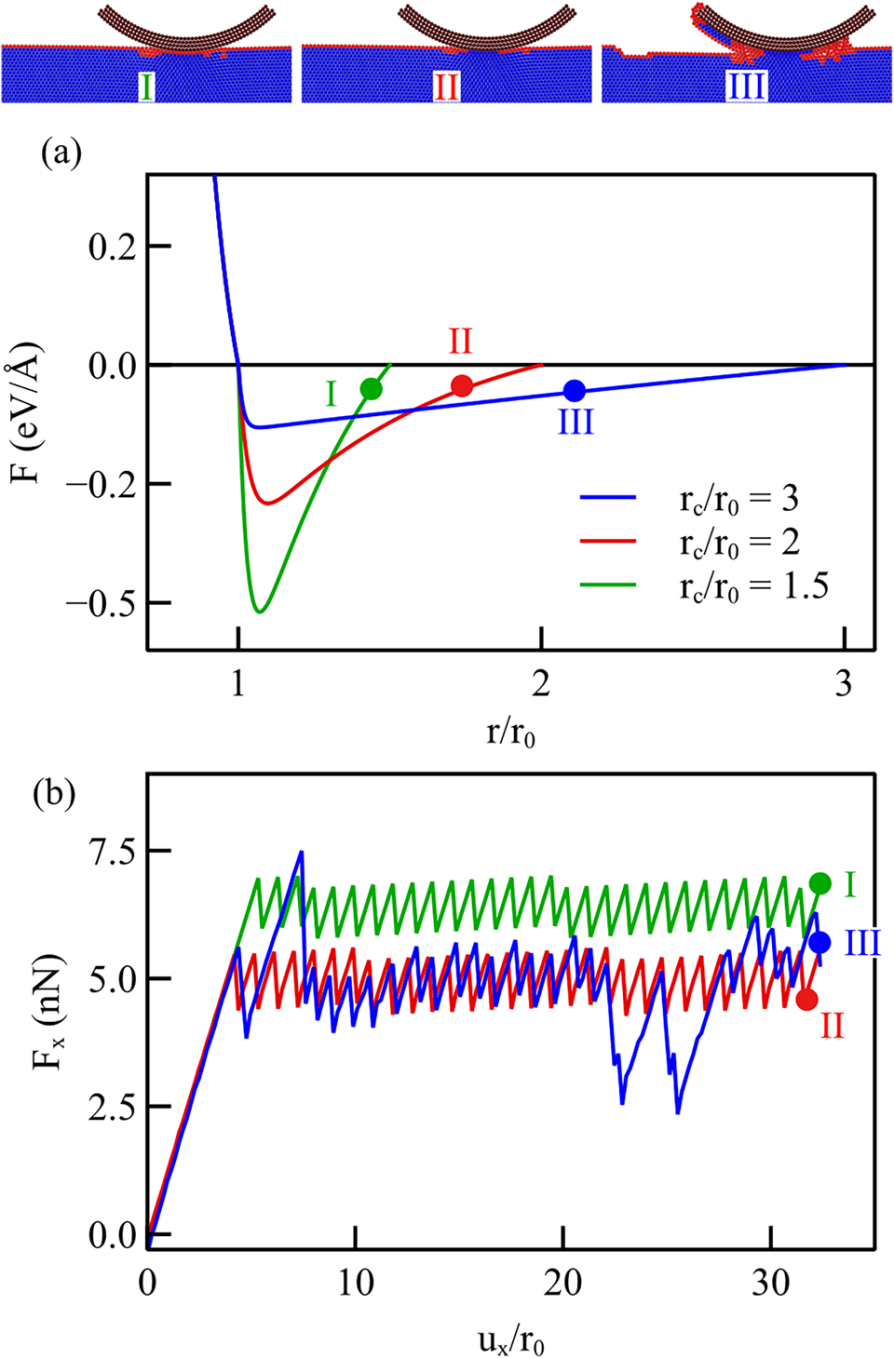
**a** Force functions with different values of the cut-off distance; **b** frictional force computed during lateral displacement of the indenter under a normal loading of $F_{y}={11.2}{\hbox {nN}}$. The insets on top represent for each interatomic potential the Common Neighbor Analysis of the atoms at the end of the simulation. Blue atoms have maintained their FCC structure during loading, while red atoms are out of registry

Simulations are here performed for the sliding of a cylindrical probe on a flat metal crystal under a light normal loading of $F_{y}={11.2}{\hbox {nN}}$. The interaction potentials describing the adhesion between the contacting surfaces are obtained using Eq. 1 with various cut-off distances, namely $r_\textrm{c}/r_\textrm{0}$ = 1.5, 2, and 3. The resulting force functions are presented in Fig. 4a. In these force functions, the values of the cut-off distance $r_\textrm{c}$, the maximum adhesion force $F_\textrm{max},$ and the interatomic distance at which the adhesion force is maximum $r_\mathrm{F_\textrm{max}}$ are all different. We identify the three force functions in Fig. 4a by labeling them with roman numbers in the same colors as the curves, so that they can be easily correlated to the frictional and wear mechanism they caused. Case I corresponds to the force function with larger $F_\textrm{max}$ but smaller cut-off radius ($r_\textrm{c}$/$r_\textrm{0}$=1.5); Case III to the force function with smaller $F_\textrm{max}$ and larger cut-off radius ($r_\textrm{c}$/$r_\textrm{0}$=3); and Case II corresponds to the intermediate force function. While the cylindrical probe is displaced tangentially on the flat surface by $\mathrm u_\textrm{x}$, the lateral force, which can be identified as a friction force that resists sliding, is computed as the sum of the atomic forces experienced by the indenter and presented in Fig. 4b as a function of the displacement. The average lateral force is largest for Case I, the case with larger maximum force and the smaller cut-off distance. The force–displacement curve exhibits the regular stick–slip pattern typically observed in experiments where an AFM tip is dragged on a flat surface. When decreasing the maximum force from Case I to Case II, the effect is a decrease of the average tangential force, while the stick–slip pattern appears unchanged. It is only with the force function that we denote Case III which the lateral force profile becomes irregular and with more pronounced peaks and valleys. Interestingly, Case II and III give rise to the same average lateral force, despite they differ in maximum adhesive force. We conclude therefore that there is no one-to-one correspondence between maximum adhesive force and friction coefficient.

From the insets at the top of Fig. 4, representing the crystals at the final sliding distance, it is possible to see that the force function that we indicated as Case III, which is the only one that produces an irregular lateral force–displacement profile, has induced wear. It is the only case where atoms have detached from the substrate to attach to the rear of the probe and have piled-up in front of it. Evidently the potentials denoted by Case II and III, although inducing on average the same friction force at the same applied normal load, are responsible for markedly different mechanisms: in one case only sliding in the other case wear. It is important to highlight that the force function that induces wear is the one with the smallest maximum adhesive force, but the largest cut-off radius, but most importantly the largest adhesive energy.

To conclude, we here noticed a change in the wear behavior at a certain threshold in cut-off radius: When the cut-off is smaller than three times the lattice parameter, no wear is observed; for larger values, it is observed, even if the maximum adhesive force is lower. Note that the results obtained here, only intend to show a trend, not to give threshold values for the interaction range or maximum force, as the occurrence of wear strongly depends on normal loading, as well as on the potential function describing the interaction. To try and isolate the effect of the maximum force from that of the cut-off, we will in the following section consider force functions with constant cut-off radius.

## Potential Functions with Constant Cut-off Distance

**Fig. 5:**
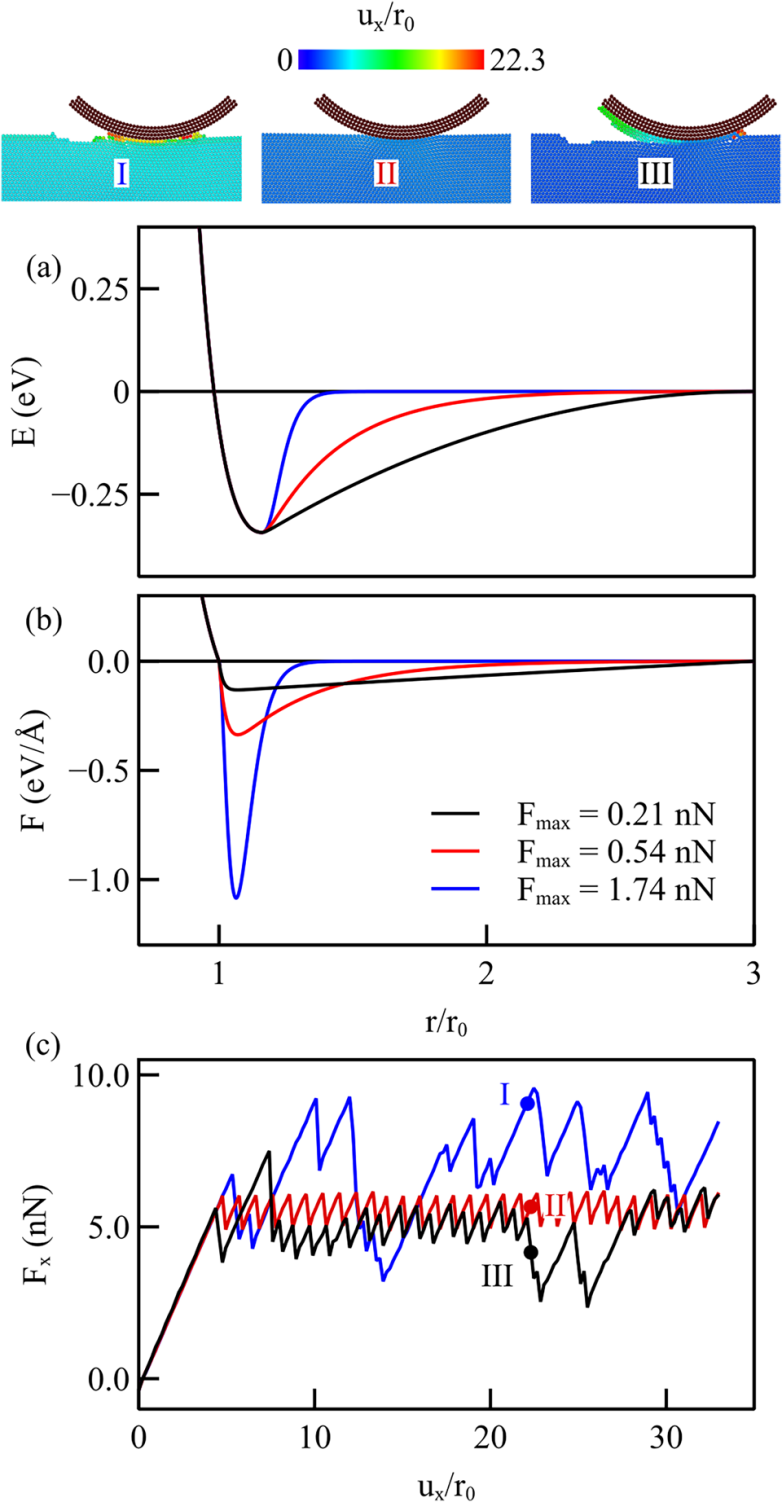
**a** Three potential functions with the same cut-off radius but different decay of the attractive potential with distance. **b** The corresponding force functions. **c** The lateral force obtained while scratching under a normal load of $F_\textrm{y} ={11.2}{\hbox {nN}}$. The figures on the right-hand side show snapshots of the distribution of the atomic lateral displacement corresponding to the three points indicated in **c** with a colored dot and a roman number

Here, we will consider interactive potentials as in Fig. 3 that have the same cut-off distance, but a different shape, which entails a different maximum adhesive force and, consequently, a different decay of the force with interatomic distance (see Fig. 5).

The cut-off distance is in all cases $r_c = 3r_0$. The maximum adhesive force $F_\textrm{max}$ is taken to occur always at $r_\mathrm{F_\textrm{max}} = 1.07r_0$ and ranges between $0.13{\text{eV}}$/Å and $1.085{\text{eV}}$/Å, corresponding to 0.21nN and 1.74nN. While these values are not specific to any couple of materials, they fall in a range of values that are realistic for actual materials. To give some examples, the attractive force between Cu and Cu is $0.233{\text{eV}}$/Å and exceeds that of Cu–C potentials [24] which is $0.10{\text{eV}}/$Å); the cut-off distance of Cu–Cu is $\sim {5.6}$Å and that of Na–Na is $\sim {16}$Å, and the curvature in the potential functions is pretty broad [25].

As in the previous sections, simulations are performed for the sliding of a rigid cylindrical probe subject to normal loading of $F_\textrm{y} ={11.2}{\hbox {nN}}$ on a flat metal crystal. The simulation starts with a very shallow indentation and the sliding follows.

The simulations are characterized by three distinct behaviors depending on the maximum adhesive force, as presented in Fig. 5, where the lateral force experienced by the probe is also presented together with snapshots of the simulations illustrating atomic displacements in the sliding direction, to capture wear. The characteristic features of the three cases are the following: (1) For the larger value of the maximum adhesive force, there is atomic wear, as can be seen by the roughness on the surface of the originally flat crystal and by the large displacement experienced by the atoms just underneath the probe; (2) For the intermediate values of the maximum adhesive force, no wear is observed. Note that in the figure, we only report one case without wear, but this happens for a range of values: also cases with $F_\textrm{max}={0.23}{\hbox {nN}}$ and ${1.05}{\hbox {nN}}$ were simulated and did not display any wear; (3) For small values of the maximum adhesive force, the crystal shows again atomic wear, with many atoms of the copper crystal detaching from the surface and attaching to the rear of the indenter. The simulations where wear is absent are characterized by a lateral force that displays the typical stick–slip behavior and is on average nearly flat, with an average value close to $F_\textrm{ave}={3.47}{\hbox {eV}}={5.56}{\hbox {nN}}$. For the simulation having the larger maximum adhesive force (case I), a larger average friction force is observed, as well as a very irregular pattern, with long stick periods, indicating that there is a significant engagement between indenter and crystal. This is confirmed by the snapshot representing the lateral displacement of the atoms: all the atoms in the crystal have experienced lateral displacement at the increment presented in the figure, which means that the full crystal was sheared by the moving indenter.

When the maximum adhesive force is small (case III), the friction force is on average even lower than that of the simulations not displaying any wear. The lateral force curve presents the typical stick–slip behavior interspersed with larger force drops. The force drops observed both for the larger and smaller $F_\textrm{max}$ can be either caused by the relative motion of indenter and crystal, or by the relative motion of some atoms of the crystal adhering to, or scratched away by, the indenter with respect to the rest of the crystal.

**Fig. 6:**
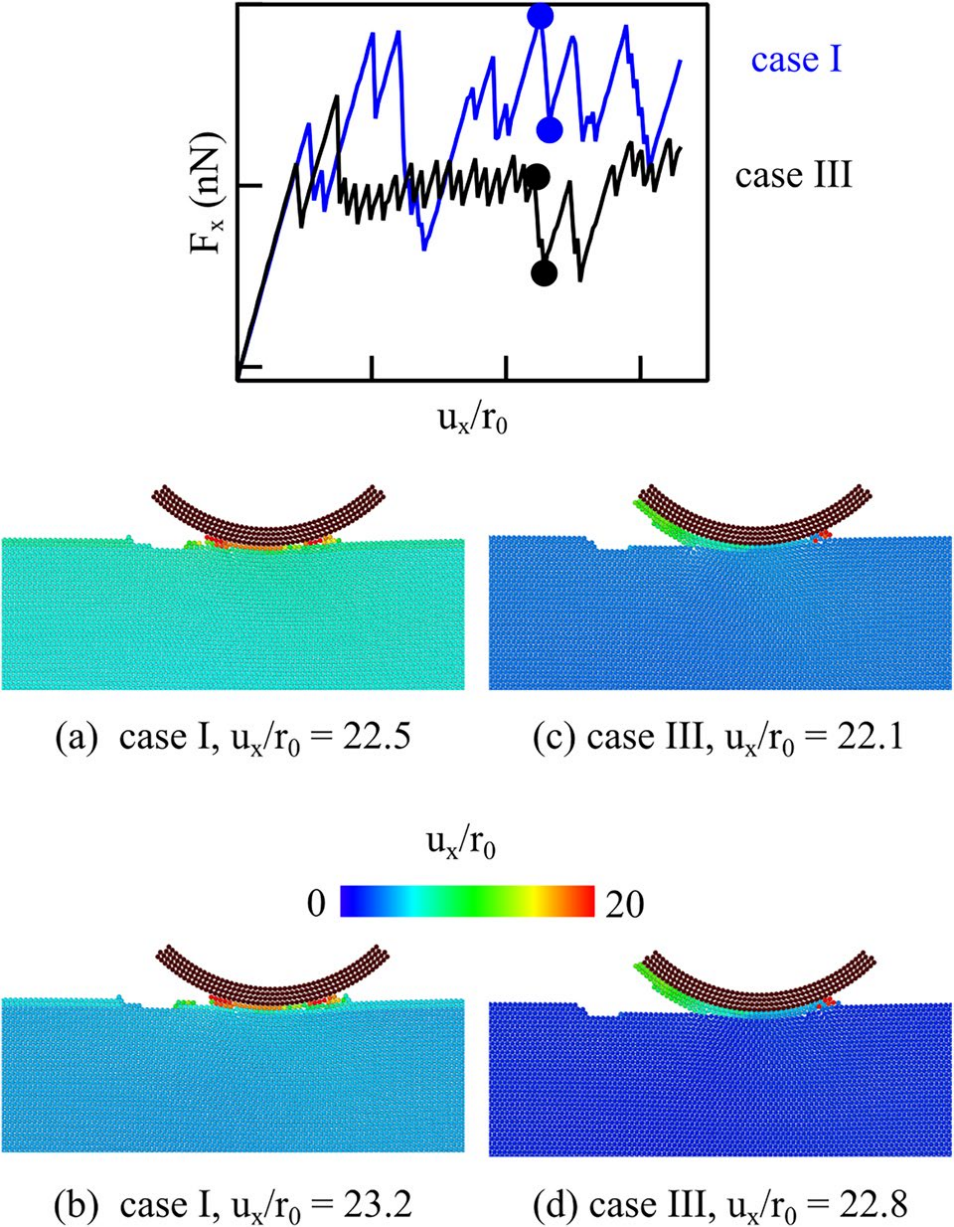
Atomic displacement in *x*-direction for case I (**a**, **b**) and case III (**c**, **d**) just before sliding and just after sliding of the probe by $\sim {6}{\hbox {nm}}$

To gain better understanding on which mechanism is active during sliding, Fig. 6 contrasts snapshots of the distribution of the lateral atomic displacement for case I and case III at a local maximum and minimum of the friction force, as indicated by the dots in the inset to Fig. 6. In both cases I and III, all atoms in the crystals are displaced laterally before sliding and go back to being less displaced after sliding. In case III, the displacement of the crystal both before and after sliding is smaller than in case I. This entails that in case I, there is a larger engagement between crystal and indenter before sliding and that, even immediately after sliding, the attractive interaction between probe and crystal causes some deformation of the crystal, while the interaction appears much smaller in case III. When looking at the atoms just below the indenter, the most striking difference between case I and III is that in case III, there are many atoms attached to the rear of the indenter, which have presumably detached from the crystal after some sliding, considering that they have displaced less than the indenter. In case I, the atoms just below the indenter have all moved much more than the underlying crystal, and there seems to be no difference in their displacement before and after sliding. Therefore, we can conclude that in case I, the sliding occurs between the atoms just below the indenter, quite strongly attached to it, and the rest of the crystal. So it is the atoms just below the indenter that are worn away. In case III, on the contrary, apart from the few atoms at the very front of the indenter, the other atoms in contact have a displacement that is smaller than that of the crystal, both before and after sliding. This points to a different mechanism: at least part of the sliding occurs between the indenter and the crystal, as in case II.

**Fig. 7:**
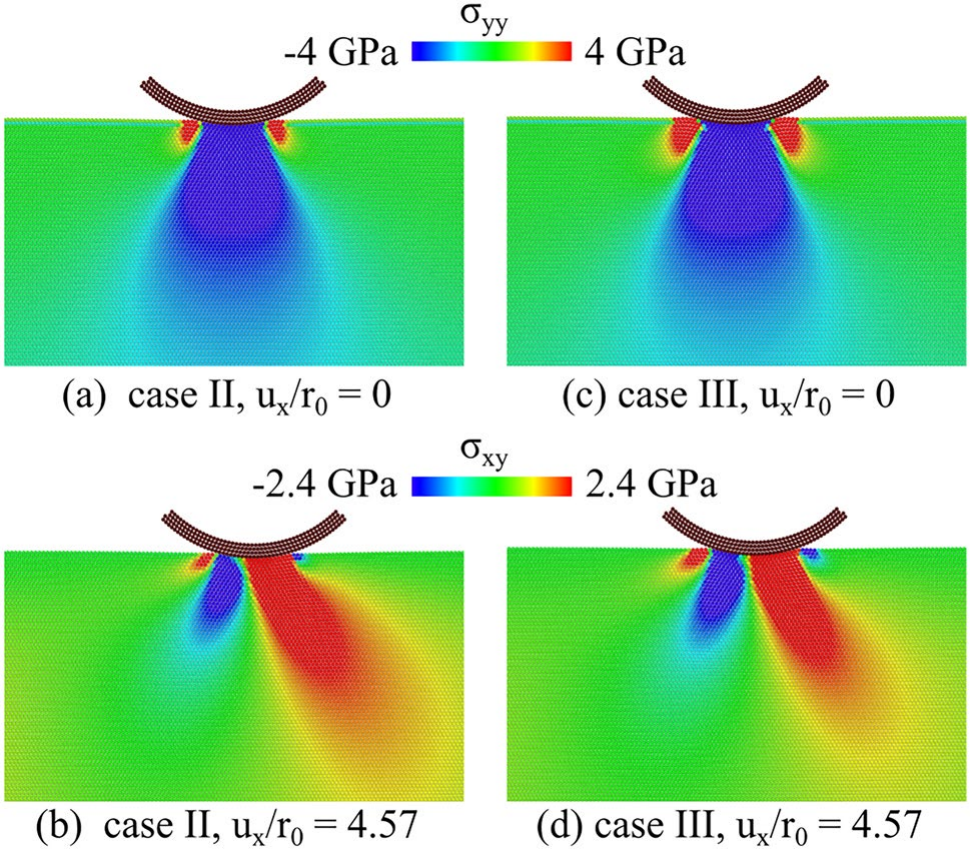
Distribution of the normal tensile stress $\sigma _\textrm{yy}$ in cases **a** II and **c** III just before sliding starts. $\sigma _\textrm{xy}$ field of cases **b** II and **d** III just before the critical shear strain of 0.5 is reached, that we used as a threshold to measure wear in case III

What remains unclear is why the simulations for case III, with the smallest force maximum, do display some wear, while the simulation for case II do not. The most likely reason for this is that, although the interatomic potential functions were selected to have the same cut-off radius, the decay from the maximum is slowest when the maximum force is smaller. This difference could results in an effectively larger range of adhesion for case III. We expect therefore that the worn atoms that attach to the rear of the contact are atoms that were less strongly bonded to the rest of the crystal in virtue of a slightly larger range of adhesion with the indenter. To check whether this is indeed the case, we present in Fig. 7a the distribution of the normal stress just after indentation, before the sliding starts, and find indeed that the tensile stress distribution is broader in case III (Fig. 7c). This is what determines a broader influence of the indenter on the crystal atoms close to it, which are, therefore, less strongly bonded to the rest of the crystal, and induces their detachment at the onset of sliding. After shearing starts and just before the first slip occurs we observe the usual butterfly pattern for the shear stress field in both cases II and III as shown in Fig. 7b and d; however, the small positive stressed region below the rear of the indenter in case III is again slightly broader than in case II. It is in correspondence of this shear stress that a dislocation nucleates in case III and leads to the occurrence of the local wear that is absent in case II.

## Atomic Wear

**Fig. 8:**
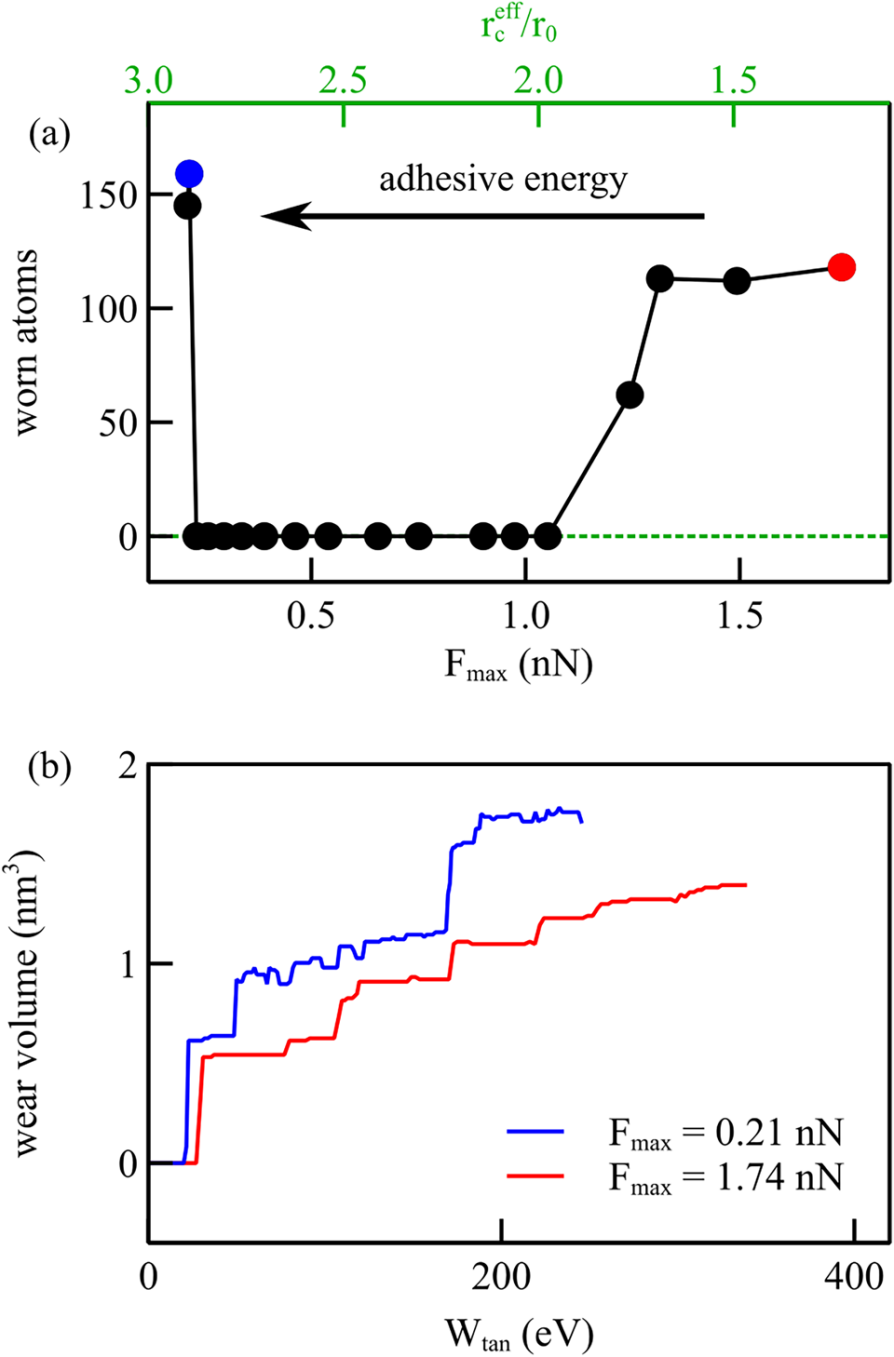
**a** Worn atoms after lightly scratching flat crystals with interaction potential functions with various $F_\textrm{max}$ and various effective interaction length. **b** Wear volume vs. tangential work for the force function with largest and smallest $F_\textrm{max}$, i.e., $F_\textrm{max} ={0.21}{\hbox {nN}}$ and $F_\textrm{max} ={1.74}{\hbox {nN}}$

Following Ma and Aghababaei [26], we identify the worn atoms as those with shear strain exceeding a critical value, which we set to be 0.5, as this threshold detects the atoms that are visibly dislodged in our simulations. Figure 8a presents the number of atoms worn away at the end of simulations performed with eighteen different potentials, all the ones we considered in this study. It is noteworthy that three different regimes can be identified and that wear is observable for force functions with either small or large values of $F_\textrm{max}$ which correspond to large or small values of the effective interaction length, here defined as the distance at which the interaction energy becomes as small as ${5 \times 10^{-4}}{\hbox {eV}}$. The adhesive energy, given by the area under the force function with $r>r_0$, increases form right to left, as the effective interaction length. One can see that the three cases considered in the previous section (indicated in the figure) that have the same cut-off radius, have in fact a very different effective interaction length and follow, thus, three different behaviors: for smaller effective interaction length but large maximum adhesive force there is wear, despite the adhesive energy is small. As previously mentioned, this type of wear occurs owing to strong interaction between probe and substrate and involves the atoms that are just below the probe, which follow closely the indenter displacement and thus detach from the substrate. This is the type of behavior one could ascribe to a dry contact. When the maximum adhesive force is reduced, as in Case II, provided that the effective interaction length remains sufficiently small to maintain the adhesive strength low, no wear is observed, as in the case of a well-lubricated contact. Wear appears again, even further decreasing the maximum adhesive force, when the effective interaction distance is sufficiently large that also the adhesive energy reaches a threshold above which significant shear strain is again observed.

In Fig. 8b, we show the relationship between wear volume and frictional work, calculated as $\int F_{x} \,dx$, for the force functions with the smallest and the largest maximum adhesive force. Less tangential work is required to wear off the same amount of atoms when the maximum adhesive force is smaller and the effective attraction distance larger. This is the case where the first significant wear event occurs after nucleation of a dislocation in the material, and is, thus, assisted by plasticity.

### Indenters with Various Radii

The amount of interaction between probe and indenter, and thus, the amount of wear, are obviously also affected by the other important length in this problem, namely the radius of the indenter. Here, we consider two additional indenters with different radii, i.e., smaller, $R={2}{\hbox {nm}}$, and larger, $R={16}{\hbox {nm}}$ than the one in the previous section, $R={8}{\hbox {nm}}$

**Fig. 9:**
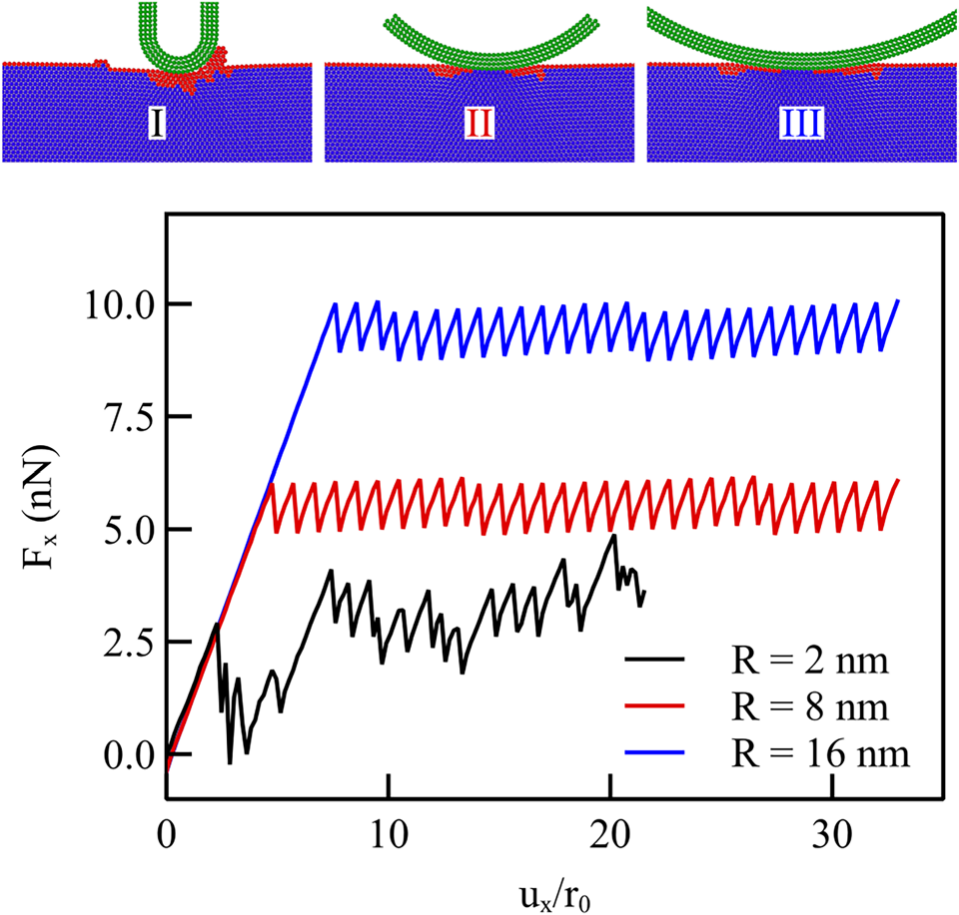
Lateral force caused by indenters with radii ${2}{\hbox {nm}}$, ${8}{\hbox {nm}}$ and ${16}{\hbox {nm}}$ and $F_\textrm{y} ={11.2}{\hbox {nN}}$. Figures on the right-hand side show the Common Neighbor Analysis (CNA) for three selected displacements of the indenters as indicated by points on the curves. Blue atoms are atoms with coordination typical of an FCC crystal, and red atoms have a non-classified coordination

Figure 9 shows the lateral force caused by the three indenters when the interaction is described by the potential function which gave no wear in the previous section, namely the one with $F_\textrm{max} ={0.54}{\hbox {nN}}$ (the red curve in Fig. 5a). In all simulations the normal load applied is $F_\textrm{y} ={11.2}{\hbox {nN}}$, which in the case of the smaller indenter radius caused a deeper indentation. Therefore, although the interaction potential function does not lead to wear for the larger indenters, it does for the smaller indenter. It is interesting to observe that the tangential force at which the indenter starts to slide is smaller when the indenter is smaller, indicating that despite the indenter has entered more deeply in the material, sliding by locally dislodging atoms is easier with such a small contact area than shearing the crystal.

### Effect of Normal Loading

**Fig. 10:**
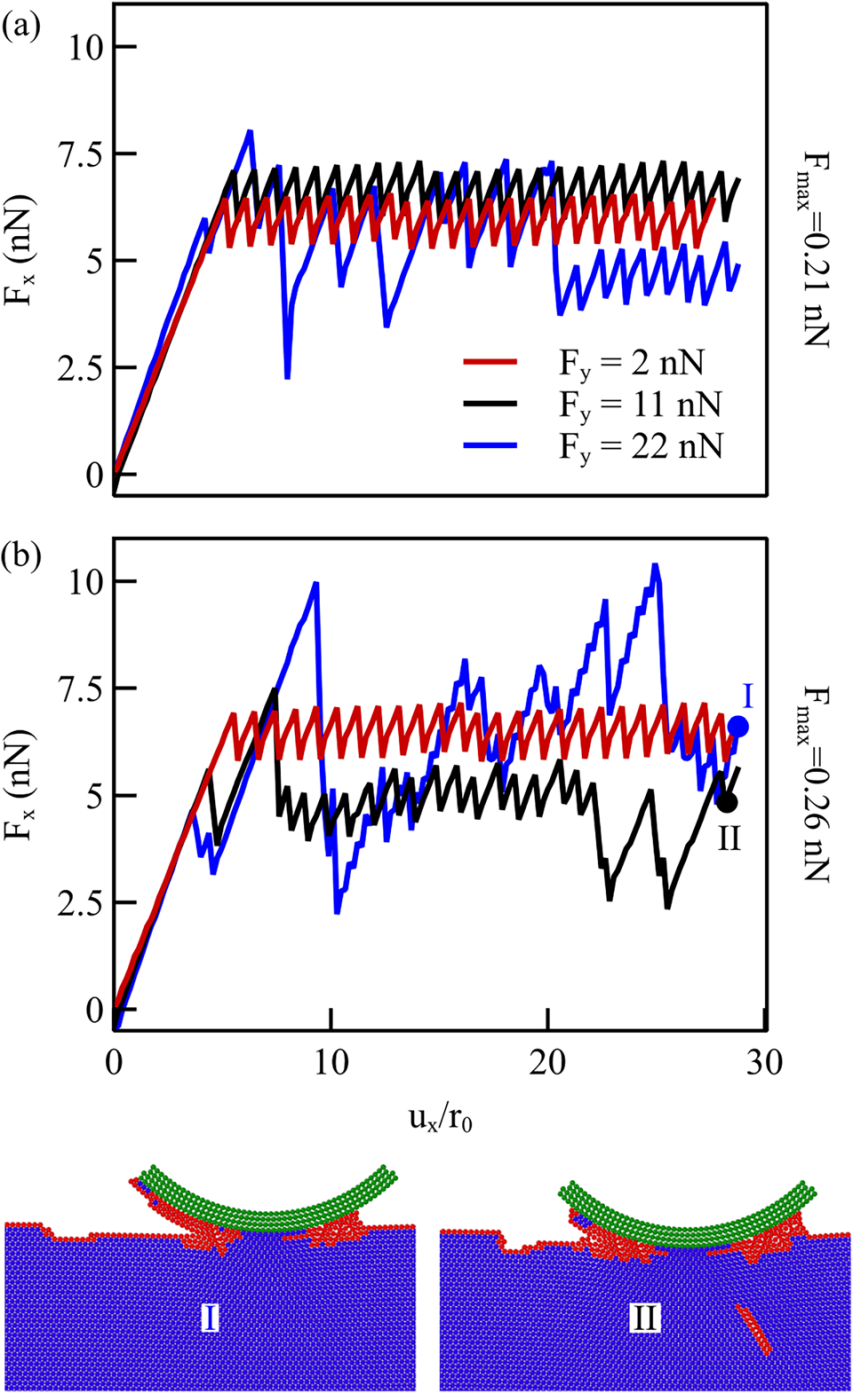
Lateral force for simulation where the probe is subject to different normal loading for potentials with **a** $F_\textrm{max} ={0.26}{\hbox {nN}}$ and **b** $F_\textrm{max} ={0.21}{\hbox {nN}}$. The insets at the bottom show two snapshots of the scratched crystal for the potential with smaller maximum force, being worn when loaded at $F_\textrm{y} ={11.2}{\hbox {nN}}$ and $F_\textrm{y} ={22.4}{\hbox {nN}}$. The colors follow the CNA, the atoms in blue are in a perfect FCC packing

It is well recognized that when increasing the normal load, wear is enhanced, since more atoms are influenced by the indenter and the interaction of the indenter and the crystal becomes stronger. If a certain normal load leads to wear, increasing the load will definitely lead to wear. It is also to be expected that a certain normal load can lead to wear with a given interatomic potential function but not with another. Despite counter-intuitive, we find that a given load causes wear for a potential with smaller maximum attractive force and does not cause wear with one with a larger maximum force and the same cut-off radius. This is demonstrated when performing scratching simulations using three increasing normal loads, namely $F_\textrm{y} ={2.4}{\hbox {nN}}$, $F_\textrm{y} ={11.2}{\hbox {nN}}$, and $F_\textrm{y} ={22.4}{\hbox {nN}}$, and two potential functions with constant cut-off and two different $F_\textrm{max}$, namely 0.26 and 0.21nN. The lateral force during displacement of the probe is shown in Fig. 10: the usual regular stick–slip pattern is the sign that no wear occurs, while an irregular pattern in the force–displacement response indicates that atoms are removed from the surface of the crystal. For the potentials with larger maximum force $F_\textrm{max}$ in Fig. 10a, an increase in normal load from $F_\textrm{y} ={2.4}{\hbox {nN}}$ to $F_\textrm{y} ={11.2}{\hbox {nN}}$ leads to a small increase in the average lateral force but no wear. A normal load of $F_\textrm{y} ={22.4}{\hbox {nN}}$ is required to observe wear. For a potential with $F_\textrm{max} ={0.21}{\hbox {nN}}$, instead (see Fig. 10b), a normal load of $F_\textrm{y} ={11.2}{\hbox {nN}}$ is sufficient to lead to wear. Similarly to what observed in previous sections, the reason why the potential with smaller force leads to wear at a smaller normal load, is that the force function decays slower to zero including in its influence range more atoms. As a consequence more atoms engage with the indenter, causing larger stressed zones during shearing which induce dislocations to nucleate during scratching.

The insets at the bottom of Fig. 10 show the CNA for the atoms at final scratching distance, when loaded normally under $F_\textrm{y} ={11.2}{\hbox {nN}}$ (on the left) and $F_\textrm{y} ={22.4}{\hbox {nN}}$ (on the right) for potentials with $F_\textrm{max} ={0.21}{\hbox {nN}}$. It is apparent that wear has occurred in both cases and that although the tangential force at the final displacement is similar for the two simulations (see the dots on the force-displacement curves), increasing $F_\textrm{y}$ increases the amount of worn atoms.

## Rough Surfaces

**Fig. 11:**
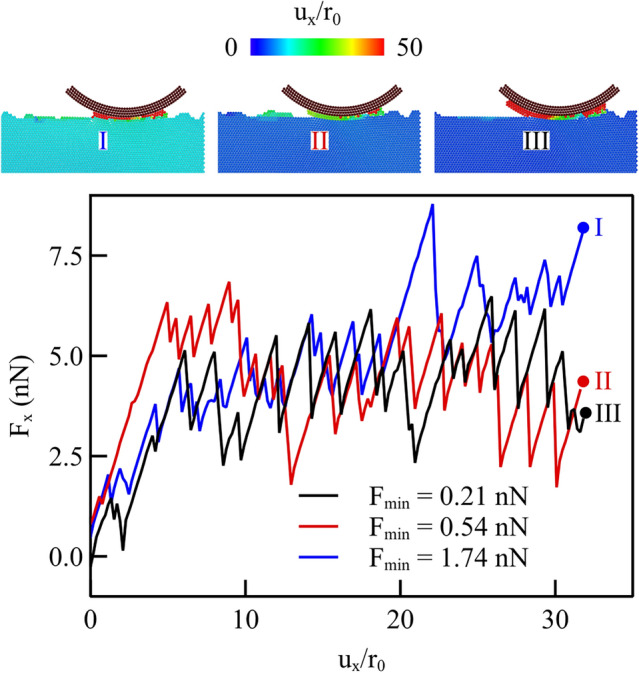
Lateral force during light scratching of rough crystals, with a normal force of $F_\textrm{y} ={11.2}{\hbox {nN}}$. The insets represent the displacement of the atoms at selected points in the curve

Simulations are here performed for a probe of radius $R={8}{\hbox {nm}}$, and the same three potential functions used in the previous section (same cut-off, different $F_\textrm{max}$, as in Fig. 5a), but now considering crystals with an atomically rough surface. The rough surface was created using a Gaussian height distribution with rms height $s_\textrm{q}={3}$Å, Hurst exponent $H=0.5$ and wavelength cut-off $q_\textrm{r}=100$. It was then relaxed using the method explained in Aramfard et al. [16]. The roughness of the three surfaces is taken to be identical before contact with the indenter. Due to the differences in the interaction potentials, however, small variation in the roughness occur already upon approach of the indenter towards the surface. The applied normal load is $F_\textrm{y} ={11.2}{\hbox {nN}}$. The variation in lateral force as a function of the lateral displacement of the probe is presented in Fig. 11. Differently from the behavior of the flat surface, the force–displacement curves for rough surfaces display a very early deviation from the elastic response, as the indenter can more easily displace atoms from the surface, which are less bonded to the crystal than the atoms on a perfectly flat surface.

Also, owing to roughness the curves do not present the typical stick–slip pattern, even in the absence of wear, as the probe experiences larger resistance to sliding when meeting a nano-hill and less resistance when meeting a nano-valley. Indeed, even the case with potential function characterized by $F_\textrm{max}={0.54}{\hbox {nN}}$ which is the one that gives no wear on flat surfaces, is here showing an irregular pattern, albeit with a rather late significant deviation from elastic behavior. The irregularity of the curve pattern is triggered by the highly non-homogeneous stress state induced by the probe on the rough surface. As a consequence, the surface atoms on the tips of the nano-hills that are poorly bounded to the rest of the crystals are easily dislodged from their position and various types of defects nucleate, including dislocations that are generally reabsorbed by the surface when the probe glides away from the nucleation site. By contrasting Fig. 11 with Fig. 5, one can see that when the surface is rough, the differences between the lateral force curves obtained with the three potentials are less evident. On average, the lateral force for the various potentials is approximately the same, although an increase in average force occurs for the potential with larger $F_\textrm{max}$ towards the end of the scratching. At the top of the figure, snapshots are shown for the atomic displacements at the end of the simulations. The case with the lowest force is the only one where the roughness has been mostly flattened by virtue of the moving indenter. The insets prove again that even the rough crystal is sheared stronger when the $F_\textrm{max}$ is larger, as a strong engagement between probe and substrate are maintained at large $F_\textrm{max}$ also when the surface is rough.

**Fig. 12:**
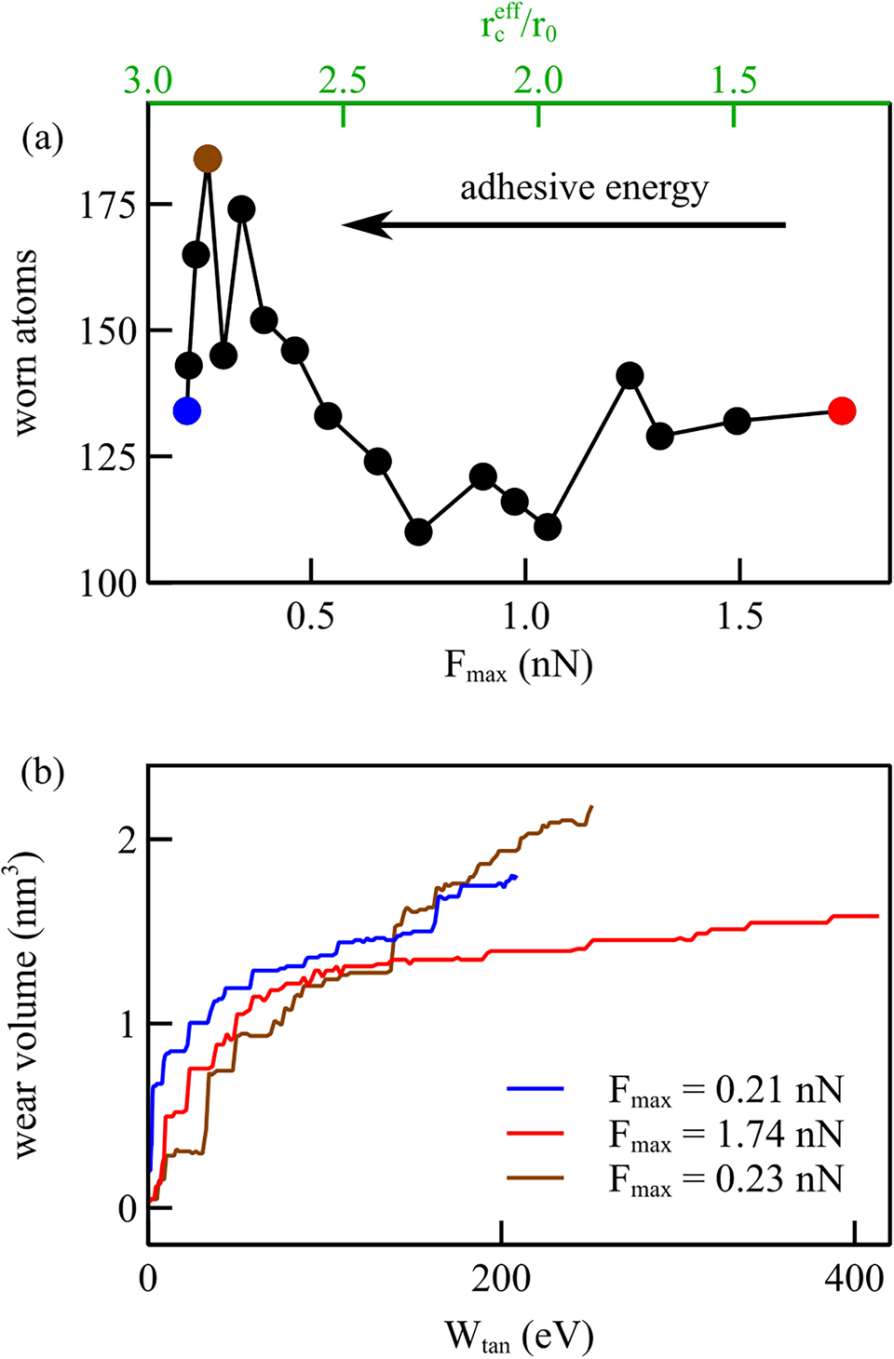
Rough surfaces: **a** worn atoms computed after scratching for various force functions, indicated according to their $F_\textrm{max}$ and effective interaction length; **b** wear volume vs. tangential work for a few selected cases, the ones with largest and smallest $F_\textrm{max}$, $F_\textrm{max} ={0.21}{\hbox {nN}}$ and $F_\textrm{max} ={1.74}{\hbox {nN}}$, and with the most worn atoms ($F_\textrm{max} ={0.23}{\hbox {nN}}$)

Also for the rough surfaces, we keep track of the worn atoms for the various force fields used in this study and present the results in Fig. 12a. Interestingly, although all force fields give rise to wear when the surfaces are rough, those with intermediate $F_\textrm{max}$ show still comparatively less wear than both cases with larger and smaller $F_\textrm{max}$. The wear volume versus the tangential work is presented in Fig. 12b for three cases, highlighted in color in Fig. 12a: in blue the case with smallest $F_\textrm{max}$, $F_\textrm{max} ={0.21}{\hbox {nN}}$, in brown the case leading to maximum wear with $F_\textrm{max} ={0.23}{\hbox {nN}}$, and in red the case with largest $F_\textrm{max}$, $F_\textrm{max} ={1.74}{\hbox {nN}}$. As in the case of flat crystals, more work is required to wear the surfaces if $F_\textrm{max}$ is larger, thus, for dryer contacts. Intriguingly, the potential marked in brown, although initially requiring more work to initiate wear, rapidly exceeds the wear volume associated with the smallest maximum force case. This suggests that while the general wear behavior on rough surfaces mirrors that observed on flat surfaces, there are more significant variations due to the way the probe interacts locally with the surface atoms.

When contrasting Fig. 12 for rough surfaces with Fig. 8 for flat surfaces, it is evident that a smaller friction energy is required to initiate wear in rough contacts, compared with flat ones. Also, when in flat contacts, the required amount of work to start wear is reached, many atoms are worn all at once, while in rough surfaces, atoms are gradually dislodged from the surfaces. For neither surfaces, we observe a linear relationship between the wear volume and the friction energy. Presumably, this is due to the small amount of wear obtained with the small normal loads considered here.

## Conclusions

In this work, we have used a tailored Morse-like interatomic potential to model the adhesive interaction between a rigid circular probe first lightly indenting, and subsequently sliding on top of a copper single crystal. The adhesive part of the interatomic potential is modified such as to vary the cut-off and/or the maximum adhesive force, and mimic a counterbody with generic adhesive properties. A large adhesive force can be linked to dry friction while a small adhesive force to wet friction. When the interatomic potentials are selected such as to have various cut-off radii, they also have various values for the maximum adhesive force. Specifically, the larger the cut-off radius, the smaller the maximum force is.

To try and avoid having both cut-off and adhesive force varying simultaneously, we have created potentials with constant cut-off and various maximum force. Nevertheless, we observed that, although with a constant cut-off, the difference in decay of the various force functions with various $F_\textrm{max}$ are so significant that effectively the interaction range still varies: the steepest force functions decay very fast to near zero values. The length that matters in determining properties is, thus, the effective interatomic attraction distance, the distance at which the attraction is larger than a very small threshold value.

With all potentials used, we could distinguish three typical behaviors for flat contacts:

- when the interatomic adhesive force $F_\textrm{max}$ is large, the friction force is on average large, and even if the effective interatomic attraction distance and, thus, the adhesive energy are small, wear occurs.
- when both the interatomic adhesive force and attraction distance are small, wear does not occur, and the friction force presents the typical stick–slip behavior during sliding.
- even if the interatomic adhesive force is small and, thus, the frictional force is small, provided the interatomic attraction range and, thus, the adhesive energy are large, wear occurs.

Although wear occurs both for large $F_\textrm{max}$ and small $F_\textrm{max}$, the amount of frictional work required to wear off the same volume of atoms is larger when $F_\textrm{max}$ is large, as the wear volume is very similar while the frictional force is larger. This means that in this work, we did not find a direct correlation between wear volume and friction energy, as observed for instance in [24, 26, 28, 29]. Presumably, this is caused partly by the very small applied normal load and wear volume considered in our work, partly by the absence of heat dissipation.

When the simulations are performed on crystals with an atomically rough surface, instead of a flat surface, the difference between the effect of using different potential functions on the friction force is much less promounced, as the atoms at the surface are poorly attracted to the rest of the crystal and easier to be dislodged. Similarly, also the effect on wear is less evident, as even the force fields with intermediate adhesive force and interaction distance, which do not show any wear when flat, display atomic wear when rough, albeit less than cases with larger and smaller adhesive force.

To conclude, the simulations show that the range of attractive interaction between contacting bodies as well as the maximum adhesive force need to be selected with great care in atomistic simulations, as they control the frictional and wear behavior of metallic surfaces. While we have here focused on force fields where the interatomic maximum force would increase at the expense of the interaction range and viceversa, one could obviously have them decrease together and thus transition from a dryer contact with a larger adhesive energy to a more lubricated contact with a smaller adhesive energy.

A potential function, as the one presented in this work can be of assistance when searching for a material with appropriate tribological properties for a given application. Moreover, this class of potential functions can be used in macro-scale simulations, when the exact interaction between the contacting bodies is unknown.

## Data Availability

The datasets generated during and/or analyzed during the current study are available from the corresponding author on reasonable request.

## References

[1] Archard, J.F.: Contact and rubbing of flat surfaces. J. Appl. Phys. **24**, 981 (1943) 10.1063/1.1721448

[2] Holms, R.: Electrical Contacts, pp. 232–242. Springer, Berlin (1976)

[3] Chung, K.H., Kim, D.E.: Method for characterizing nanoscale wear of atomic force microscope tips. Tribol. Lett. **15**, 135–144 (2003) 10.1023/A:1024457132574

[4] Liu, J., Notebohm, J.K., Carpick, R.W., Turner, K.T.: Fundamental investigation of micro wear rate using an atomic force microscope. ACS Nano **4**, 3763–3772 (2010) 20575565 10.1021/nn100246g

[5] Jacobs, T.B.D., Carpick, R.W.: Nanoscale wear as a stress-assisted chemical reaction. Nat. Nanotechnol. **8**, 108–112 (2013) 23353678 10.1038/nnano.2012.255

[6] Merkle, A.P., Marks, L.D.: Liquid-like tribology of gold studied by in situ TEM. Wear **265**, 1864–1869 (2008) 10.1016/j.wear.2008.04.032

[7] Sato, T., Ishida, T., Jalabert, L., Fujita, H.: Real-time transmission electron microscope observation of nanofriction at a single Ag asperity. Nanotechnology **23**, 505701 (2012) 23164958 10.1088/0957-4484/23/50/505701

[8] Sorensen, M.R., Jacobsen, K.W., Stoltze, P.: Simulations of atomic-scale sliding friction. Phys. Rev. B **53**, 2102–2113 (1996) 10.1103/physrevb.53.2101 9983674

[9] Zhong, J., Shakiba, R., Adams, J.B.: Molecular dynamics simulations of severe adhesive wear on a rough aluminum substrate. J. Phys. D Appl. Phys. **46**, 055307 (2013) 10.1088/0022-3727/46/5/055307

[10] Bian, J.J., Nicola, L.: On the lubrication of rough copper surfaces with graphene. Tribol. Int. **156**, 106837 (2021) 10.1016/j.triboint.2020.106837

[11] Bian, J.J., Nicola, L.: Lubrication of rough copper with few-layer graphene. Tribol. Int. **173**, 107621 (2022) 10.1016/j.triboint.2022.107621

[12] Aghababaei, R., Warner, D.H., Molinari, J.F.: Critical length scale controls adhesive wear mechanisms. Nat. Commun. **7**(1), 1–8 (2016) 10.1038/ncomms11816 PMC4897754 27264270

[13] Rabinowicz, E.: The effect of size on the looseness of wear fragments. Wear **2**, 4–8 (1958) 10.1016/0043-1648(58)90335-1

[14] Morse, P.M.: Diatomic molecules according to the wave mechanics. II. Vibrational levels. Phys. Rev. **34**, 57–64 (1929) 10.1103/PhysRev.34.57

[15] Alhafez, I.A., Urbassek, H.M.: Influence of tip adhesion on nanoindentation and scratching. Model. Simul. Mater. Sci. Eng. **27**, 065014 (2019) 10.1088/1361-651X/ab27ed

[16] Aramfard, M., Pérez-Ràfols, F., Nicola, L.: A 2D dual-scale method to address contact problems. Tribol. Int. **171**, 107509 (2022) 10.1016/j.triboint.2022.107509

[17] Daw, M.S., Baskes, M.I.: Embedded-atom method: derivation and application to impurities, surfaces, and other defects in metals. Phys. Rev. B **29**(12), 6443 (1984) 10.1103/PhysRevB.29.6443

[18] Mishin, Y., Mehl, M.J., Papaconstantopoulos, D.A., Voter, A.F., Kress, J.D.: Structural stability and lattice defects in copper: Ab initio, tight-binding, and embedded-atom calculations. Phys. Rev. B **63**(22), 224106 (2001) 10.1103/PhysRevB.63.224106

[19] Shilkrot, L.E., Miller, R.E., Curtin, W.A.: Multiscale plasticity modeling: coupled atomistics and discrete dislocation mechanics. J. Mech. Phys. Solids **52**(4), 755–787 (2004) 10.1016/j.jmps.2003.09.023

[20] Rafii-Tabar, H., Hua, L., Cross, M.: A multi-scale atomistic-continuum modelling of crack propagation in a two-dimensional macroscopic plate. J. Phys.: Condens. Matter. **10**(11), 2375–2387 (1998)

[21] Luan, B., Robbins, M.O.: Hybrid atomistic/continuum study of contact and friction between rough solids. Tribol. Lett. **36**(1), 1–16 (2009) 10.1007/s11249-009-9453-3

[22] Thompson, A.P., Plimpton, S.J., Mattson, W.: General formulation of pressure and stress tensor for arbitrary many-body interaction potentials under periodic boundary conditions. J. Chem. Phys. **131**(15), 154107 (2009) 20568847 10.1063/1.3245303

[23] Maekawa, K., Itoh, A.: Friction and tool wear in nano-scale machining-a molecular dynamics approach. Wear **188**(1–2), 115–122 (1995) 10.1016/0043-1648(95)06633-0

[24] Fang, T.H., Weng, C.I.: Three-dimensional molecular dynamics analysis of processing using a pin tool on the atomic scale. Nanotechnology **148**(3), 1–11 (2000)

[25] Lim, T.C., Udyavara, R.A.: Relations between Varshni and Morse potential energy parameters. Central European J. Phys. **7**, 193–197 (2009)

[26] Ma, L., Aghababaei, R.: On the effect of adhesive strength and scratching depth on material transfer during nanoscale scratching. Tribol. Lett. **70**(1), 26 (2022) 10.1007/s11249-021-01558-z

[27] Zhao, K., Aghababaei, R.: Interfacial plasticity controls material removal rate during adhesive sliding contact. Phys. Rev. Mater. **4**(10), 103605 (2020) 10.1103/PhysRevMaterials.4.103605

[28] Ramalho, A., Miranda, J.C.: The relationship between wear and dissipated energy in sliding systems. Wear **260**(4–5), 361–367 (2006) 10.1016/j.wear.2005.02.121

[29] Fouvry, S.: Friction energy wear approach. In: Elsevier Series on Tribology and Surface Engineering, Fretting Wear and Fretting Fatigue, pp. 87–117. Elsevier, Amsterdam (2023)

